# Insights into Antiviral Properties and Molecular Mechanisms of Non-Flavonoid Polyphenols against Human Herpesviruses

**DOI:** 10.3390/ijms232213891

**Published:** 2022-11-11

**Authors:** Sherif T. S. Hassan, Miroslava Šudomová, Alena Mazurakova, Peter Kubatka

**Affiliations:** 1 Department of Applied Ecology, Faculty of Environmental Sciences, Czech University of Life Sciences Prague, Kamýcká 129, 165 00 Prague, Czech Republic; 2 Museum of Literature in Moravia, Klášter 1, 664 61 Rajhrad, Czech Republic; 3 Department of Medical Biology, Jessenius Faculty of Medicine, Comenius University in Bratislava, 03601 Martin, Slovakia; 4 Biomedical Centre Martin, Jessenius Faculty of Medicine in Martin, Comenius University in Bratislava, Mala Hora 4D, 03601 Martin, Slovakia

**Keywords:** polyphenols, antiviral activity, herpes simplex virus, HSV-1, HSV-2, human cytomegalovirus (HCMV), varicella-zoster virus (VZV), Epstein–Barr virus (EBV), Kaposi sarcoma-associated herpesvirus (KSHV), non-flavonoid polyphenols, natural products

## Abstract

Herpesviruses are one of the most contagious DNA viruses that threaten human health, causing severe diseases, including, but not limited to, certain types of cancer and neurological complications. The overuse and misuse of anti-herpesvirus drugs are key factors leading to drug resistance. Therefore, targeting human herpesviruses with natural products is an attractive form of therapy, as it might improve treatment efficacy in therapy-resistant herpesviruses. Plant polyphenols are major players in the health arena as they possess diverse bioactivities. Hence, in this article, we comprehensively summarize the recent advances that have been attained in employing plant non-flavonoid polyphenols, such as phenolic acids, tannins and their derivatives, stilbenes and their derivatives, lignans, neolignans, xanthones, anthraquinones and their derivatives, curcuminoids, coumarins, furanocoumarins, and other polyphenols (phloroglucinol) as promising anti-herpesvirus drugs against various types of herpesvirus such as alpha-herpesviruses (herpes simplex virus type 1 and 2 and varicella-zoster virus), beta-herpesviruses (human cytomegalovirus), and gamma-herpesviruses (Epstein–Barr virus and Kaposi sarcoma-associated herpesvirus). The molecular mechanisms of non-flavonoid polyphenols against the reviewed herpesviruses are also documented.

## 1. Introduction

**Figure 1:**
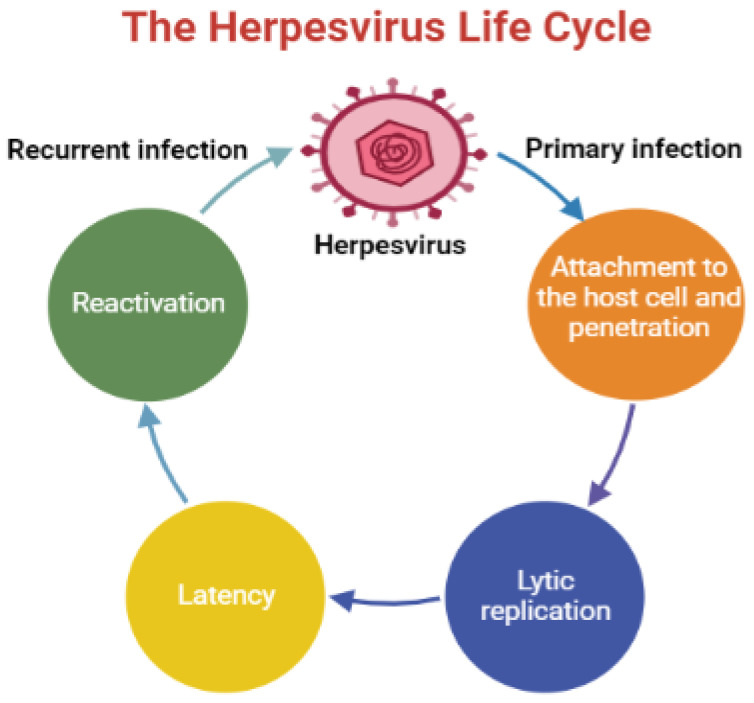
An illustration displays an overview of the herpesvirus life cycle.

Human herpesviruses (HHVs) are infectious DNA viruses that belong to the family of *Herpesviridae* with the capacity to establish lifelong latent infections, which undergo periodic reactivation [1]. These viruses induce a broad spectrum of ailments, ranging from frequent cold sores to cancer, and remain a major cause of morbidity and mortality, particularly in immunocompromised patients [2]. HHVs are categorized into three subfamilies, *Alphaherpesvirinae*, which includes herpes simplex virus type 1 and type 2 (HSV-1 and HSV-2) and varicella-zoster virus (VZV or HHV-3) [3,4] and *Betaherpesvirinae* includes human cytomegalovirus (HCMV or HHV-5), HHV-6, and HHV-7 [5]. *Gammaherpesvirinae* subfamily consists of two tumor viruses, Epstein–Barr virus (EBV or HHV-4) and Kaposi sarcoma-associated herpesvirus (KSHV or HHV-8) [6]. Over millions of years, HHVs have developed various mechanisms to infect their hosts and modulate their genetic materials [4]. The infection begins by attaching the virus to the host cell, specifically, to the binding receptors via several viral glycoproteins (the heterodimer gH–gL and the viral fusion protein gB), which are placed on the surface of the virion [7,8]. The life cycle of HHVs comprises two critical phases: lytic infection and latent infection. During lytic infection, the virus replicates and produces several progeny virions, and then travels to the latent site (in this stage, the virus is inactive) to form latent infection [9,10]. Subsequently, the virus reactivates once the host’s immune system is weakened by diverse physiological and environmental factors that adversely affect the immune system. The reactivation process depends on the nature of the latently infected cell [11,12]. HHV infections are usually asymptomatic, and the symptoms occur once the immune system is compromised [13]. An overview of the herpesvirus life cycle is shown in Figure 1.

The current effective medical treatment of HHVs is based on acyclovir and other related antiviral medications that target viral DNA polymerases. The overuse of these drugs has led to the developing of the problem of drug resistance, leading to unsuccessful treatment efficacy [14,15]. Moreover, acyclovir and related nucleoside or nucleotide analogs do not cure herpes infections but reduce the severity and frequency of symptoms [16]. Therefore, the search for new sources such as natural products that provide effective drugs with the ability to inhibit the herpesvirus at diverse stages of the life cycle with no toxicity and resistance is urgently needed [17,18]. 

Consequently, in this article, we comprehensively review the recent investigations that have been performed on the use of plant non-flavonoid polyphenols as promising antiviral agents against HHVs such as alpha-herpesviruses, beta-herpesviruses, and gamma-herpesviruses. To understand how these compounds work as antiviral drugs, we showcase their mechanisms of action at molecular and cellular levels against the reviewed herpesviruses. The effective concentrations or doses are also highlighted. 

Several online databases such as Web of Science Core Collection, PubMed, Scopus, SciFinder, Google Scholar, Embase, and ScienceDirect were used during the literature search utilizing proper keywords that characterize antiviral activities of plant polyphenols (excluding flavonoids) against HHVs. Studies that have been published (in the English language) in the years from 2017 to July 2022 were selected to collect the required data. However, limited studies published before 2017 have been included to support or criticize the validity of the data obtained from recent investigations. 

## 2. An Overview of Polyphenols and Their Health Benefits as Antivirals

Polyphenols are a superfamily of a large group of phytochemicals that naturally occur in plants with different structures and properties. Their primary function is to protect plants from pathogenic infections, UV radiation damage, herbivores, and promote the development process [19,20]. Protection against other abiotic stresses, including salinity, drought, toxic metals, and extremely low or high temperatures, has been reported as a significant role of polyphenols in plants [21,22]. Polyphenols are present in many commonly consumed vegetables, fruits, herbs, and grains and their multiple therapeutic activities make them important factors contributing to maintaining the health of the organism [23,24]. The amount of polyphenols in plants varies and depends on several factors, including the plant maturation state, growing, and storage conditions, and the extraction process [25,26]. Information regarding polyphenols classification and their chemical structures has been well-documented in a recently published review article by Durazzo et al. [21]. Readers who are interested in this information can refer to the recommended reference. 

In various preclinical and clinical studies, polyphenols have proven therapeutic efficacy for many diseases including viral infections [24]. It has been shown that polyphenols, including their subclass flavonoids, inhibit the replication of diverse human DNA and RNA viruses by various mechanisms of action at different molecular levels [27,28].

## 3. Antiviral Properties of Non-Flavonoid Polyphenols against Alpha-Herpesviruses

Human alpha-herpesviruses are a group of infectious DNA viruses that includes important human pathogens such as HSV-1, HSV-2, and VZV. HSV-1 is the causative agent of herpes labialis (the symptoms are recognized as cold sores) with the capacity to generate genital herpes (a sexually transmitted infection), while HSV-2 induces primarily genital herpes characterized by the presence of ulcerative or vesicular lesions [29,30]. VZV causes both varicella (chickenpox) by initial infection and herpes zoster (shingles) by reactivation from latency [31,32]. Human alpha-herpesviruses are transmitted in different ways and direct contact with an infected individual is the most common mode. Their infections are usually asymptomatic, and slight symptoms might appear that go unnoticed in some cases [33,34]. 

In this section, we critically discuss the recent experiments that feature the anti-herpesvirus properties of polyphenols (excluding flavonoids) against HSV-1, HSV-2, and VZV. The molecular mechanisms and effective concentrations or doses are also highlighted. 

### 3.1. Phenolic Acids

In an in vitro assay, ginkgolic acid, a phenolic compound detected in the leaves and fruits of *Ginkgo biloba*, was observed to inhibit the replication of HSV-1 in infected HEp-2 and 293T cells at various concentrations ranging from 2.5 to 50 µM. The results showed that ginkgolic acid has blocked HSV-1 infection by inhibiting viral protein synthesis such as immediate early (ICP27), early (ICP8), and late (US11) proteins. Moreover, ginkgolic acid has effectively repressed viral progeny production [35]. In another work, an extract of *Ginkgo biloba* containing ginkgolic acid (<5 ppm) possessed anti-HSV-1 action before viral adsorption to the cell surface, interrupted the viral structure, and impeded HSV-1 virion entry. The anti-HSV-1 action was proposed to be linked to the presence of ginkgolic acid [36]. An additional study has also explored the antiviral activity of ginkgolic acid against HSV-1 skin infection in BALB/cJ mice. At an effective dose of 10 mM prepared in 2.5% hydroxyethyl cellulose gel (administered twice daily for 14 days), ginkgolic acid was found to significantly decrease mortality, infection score, and durations of HSV-1 cutaneous infection in a zosteriform model in BALB/cJ mice. It also lessened the replication of an acyclovir-resistant strain of HSV-1 in Vero cells at a concentration of 10 µM [37].

Protocatechuic acid from *Hibiscus sabdariffa* L. was identified to deactivate the replication of HSV-2 DNA in Vero cells with a 50% effective concentration (EC_50_) value of 0.92 µg/mL. Inhibition of the replication of HSV-2 DNA was mentioned as a mechanism of action [18]. 

In a molecular docking study, Todorova and colleagues [38] showed that trans-ferulic acid, gentisic acid, vanillic acid, syringic acid, and gallic acid extracted from the phenolic fraction of *Graptopetalum paraguayense* E. Walther might inhibit HSV-1 DNA polymerase, a critical enzyme required for HSV replication. The test compounds showed the capacity to effectively bind to the active site of the enzyme, leading to possible inhibition of HSV-1 activity.

AbouAitah et al. [39] have recently designed a nano-formulation composed of ellagic acid and functionalized zinc oxide nanoparticles (ZnO NPs) with enhanced anti-HSV-2 activity. The hybrid nano-formulation, ZnO NPs, and ellagic acid selectively inactivated HSV-2 DNA replication with 50% inhibitory concentration (IC_50_) values of 3.6, 5.4, and 4.0 µg/mL, respectively, and selectivity index (SI) 57.5, 41.2, and 28.1, respectively.

Treatment of HSV-1-infected Vero cells with a polyphenol-rich extract from *Solanum melongena* L. for 24 h after viral adsorption hindered the viral replication with an IC_50_ value of 83.4 µg/mL by targeting the viral gB expression. The presence of chlorogenic acid, caffeic acid, and vanillic acid, along with other polyphenolic compounds identified in the extract, was proposed to be correlated to the induced anti-HSV-1 properties [40]. In another work, Langland et al. [41] evaluated the anti-HSV effects of phenolic acids when paired with metal and inorganic ions. Metal chelates of caffeic acid (addition of cations such as Fe^3+^ and anionic molecules such as molybdate and phosphate) increased antiviral activity upwards of 100-fold. Specifically, caffeic acid chelates exerted the best effects against HSV-1 and HSV-2. Besides, due to their action on extra-cellular processes such as inhibition of virion binding to the cell, the authors concluded that the potential of adding caffeic acid chelates to existing medications such as acyclovir could provide more efficient management of HSV infections.

### 3.2. Tannins and Their Derivatives

In an ethnopharmacological study, chebulagic and chebulinic acids were isolated from the fruits of *Terminalia chebula* Retz. and were further examined for their anti-HSV-2 properties. Both compounds inhibited the viral activity with IC_50_ values of 1.41 and 0.06 µg/mL, respectively. Moreover, in the post-infection plaque reduction test, both molecules suppressed the replication of HSV-2 with IC_50_ values of 31.84 and 8.69 µg/mL, respectively. The study outcome suggests that both compounds might prevent the attachment and penetration of the virus to Vero cells [42].

The antiherpetic action of geraniin (extracted from the leaves of *Spondias mombin* L.) against HSV-1 was studied in a combined in vitro and in silico experiment. The in vitro results demonstrated that geraniin at a concentration of 20.40 µg/mL exhibited virucidal action via blocking viral attachment. The mechanism of action was predicted using a molecular docking analysis via targeting the glycoprotein gB of the HSV-1 surface [43].

Tannic acid (TA), a plant-derived hydrolyzable tannin with five digalloyl units connected with one glucose molecule, has recently been designed by Szymańska et al. [44] in mucoadhesive gelling delivery systems using silver nanoparticles modified with tannic acid (TA-AgNPs) for possible enhanced HSV therapy. The authors have demonstrated in vitro the ability of TA-AgNPs-based hydrogels (at concentrations of 25 or 50 ppm) to potently inhibit HSV-1 and HSV-2 replications. The mechanism of HSV-1 inhibition was revealed by blocking the viral attachment to the immortal human keratinocyte cell line by interfering with gC and gB glycoproteins, while the anti-HSV-2 activity was mediated by hindering viral attachment and penetration. Additionally, treatment of a murine model of HSV-2 genital infection with TA-AgNPs-based hydrogels (at a concentration of 25 ppm) has successfully prevented vaginal HSV-2 transmission. 

In an animal study, another research team [45] provided interesting results in which treatment of an HSV-2-infected mouse model (with primary and recurrent vaginal infections) with TA-AgNPs (size 33 nm) improved the anti-HSV-2 immune response by boosting a virus-specific cellular and humoral response with activation of B cells.

Selected groups of natural tannins such as ellagitannins and gallotannin-type compounds have been assayed for their antiviral effects on HSV-1 replication performed on monolayer cultures of Madin-Darbey bovine kidney (MDBK) cells. Based on the calculation of selectivity index (SI; CC_50_/IC_50_), ellagitannins such as epiacutissimin B (SI > 60.6), epiacutissimin A (SI > 55.5), acutissimin A (SI > 34.8), and mongolicain (SI > 32.5) demonstrated greater inhibition of HSV-1 replication than gallotannin-type compounds such as 1,2,3,4,5-penta-*O*-digalloyl-*β*-*D*-glucopyranose (SI > 35.7), 1,2,3,4,5-penta-*O*-digalloyl-*α*-*D*-glucopyranose (SI > 28.5), tannic acid (SI > 25), and α/β-3-*O*-digalloyl-*D*-glucopyranose (1:1 mixture; SI = 15.6). The non-nucleoside structure of the test compounds was suggested to be accountable for the anti-HSV-1 properties. The mechanisms of action have been proposed to be related to the ability to target HSV-1 glycoproteins. [46]. In another experiment, the combination of C-glucosidic ellagitannins castalagin and vescalagin (isolated from *Quercus robur*) with acyclovir applied against acyclovir-resistant HSV-1 possessed a much stronger synergistic effect compared with the effect detected against acyclovir-resistant HSV-2 with IC_50_ values ranging from 0.04 to 0.46 µM [47]. 

Punicalagin, an ellagitannin isolated from *Punica granatum* (pomegranate), demonstrated 100% anti-HSV-2 activity determined at 31.25 µg/mL and showed an inhibitory effect equivalent to the standard drug acyclovir. The mechanism of action was predicted via targeting HSV-2 protease using molecular docking analysis [48]. Likewise, Houston et al. [49] revealed that the virucidal activity of pomegranate extract and punicalagin against HSV-1 can be potentiated when co-administered with zinc ions.

Pentagalloylglucose (1,2,3,4,6-penta-*O*-galloyl-*β*-*D*-glucose; PGG), a bioactive gallotannin extracted from *Elaeocarpus sylvestris*, has significantly suppressed VZV replication with an IC_50_ value of 14.67 µg/mL. The anti-VZV mechanism of PGG was disclosed via inhibiting VZV-induced c-Jun N-terminal kinase (JNK) activation and expression of VZV-IE62 protein [50].

### 3.3. Xanthones 

Mangiferin, a bioactive compound of *Mangifera indica* with C-glycosylxanthone structure, has been examined in vitro and in vivo for its anti-herpesvirus properties against HSV-1. Mangiferin in vitro suppressed the replication of two HSV-1 strains (ACV-resistant HSV-1 (AR-29) and sensitive (KOS)) with IC_50_ values of 2.9 and 3.5 µg/mL, respectively, with a suggested mechanism that affects viral particles. Furthermore, treatment of Balb/c mice with mangiferin topical formulation (0.7%) improved the healing course by reducing the lesions [51]. Another in vitro research has evaluated the anti-HSV-1 properties of a mixture containing mangiferin combined with a polysaccharide galactomannan derived from the tree *Dimorphandra gardneriana* (DgGmM). DgGmM repressed the replication of HSV-1 with an IC_50_ value of 287.5 µg/mL, while at post-infection treatment (1 h; 500 µg/mL), maximum inhibition was observed [52].

### 3.4. Stilbenes and Their Derivatives 

Resveratrol, a stilbene compound, has been detected in several plants, including grape vines, berries, pomegranates, pines, soybeans, legumes, and peanuts with promising antiviral properties against various human and animal viruses [53,54]. Previous preclinical studies have asserted the capacity of resveratrol to inhibit the replication of HSV-1 and HSV-2 in a dose-dependent and reversible manners via targeting viral immediate-early (IE) genes [55,56]. However, Ding et al. [57] reported in their recent investigation that resveratrol (in a dose-dependent manner) can promote HSV-2 replication and hence HSV-2 infection by boosting histone acetylation and regulating the activation of nuclear factor-κB (NF-κB). Their results also revealed that suppression of HSV-2 replication by resveratrol at a concentration of 30 µM might be achieved via repressing the activity of cyclin-dependent kinase 9 (CDK9), an enzyme necessary for HSV-2 replication. Based on the reviewed data, we can assume that resveratrol induces anti-HSV activities in a dose-dependent manner; however, the validation of its activity and mechanisms should be in-depth evaluated in further in vivo experiments. Unlike the vital role of thymidine kinase (TK; an HSV-encoded gene product and a mediator enzyme that plays a critical role in HSV replication) in HSV pathogenesis, resveratrol at concentrations of 10 and 20 µM was found to boost the bystander action induced by the HSV-TK/ganciclovir gene therapy for hepatocellular carcinoma associated with herpesvirus infections by improving connexin-mediated gap junctional communication [58]. 

Piceatannol, a resveratrol metabolite extracted from *Cassia abbreviata*, was determined to be active against HSV-1 and HSV-2 with IC_50_ values of 47.5 and 45.0 µM, respectively, by affecting viral particles. However, further studies are needed to elucidate the exact mechanism of action beyond the inhibition of viral DNA replication [59].

Tarbeeva and co-workers [60] isolated a stilbenoid with a 1,2-diketone fragment named bicoloketone from the stem bark of *Lespedeza bicolor* with potential antiherpetic activity. Treatment of HSV-1-infected Vero cells with bicoloketone has led to notable inhibition of HSV-1 replication with an IC_50_ value of 44.2 µM.

Recently, a study performed in Italy analyzed the efficacy of grape cane extract (named ‘’Greco’’ from *Vitis vinifera* L.) against HSV-1 infection. The results of high-performance liquid chromatography combined with multistage ion trap mass spectrometry (HPLC/ITMSn) analysis indicated that Greco extract at pH = 13 is rich in stilbenoids such as resveratrol C-glucoside, resveratrol, and epsilon-viniferin. Furthermore, the extract (pH = 13; 10 µg/mL) was identified to act directly with HSV-1 particles by blocking HSV-1 replication with an IC_50_ value of 0.9 µg/mL. The study outcome suggests that the anti-HSV-1 activity of the test extract is related to the presence of the detected stilbenoids [61]. 

### 3.5. Lignans and Neolignans 

Honokiol is a bioactive component of the genus *Magnolia* with multiple pharmacological actions. Its antiviral effect on HSV-1 infection has been disclosed by Liu and colleagues [62] with an IC_50_ value of 10.51 µg/mL. Its mechanism of action against HSV-1 is related to inhibiting the virus replication, ICP27 and VP16 gene expressions, and viral progeny production. Moreover, honokiol (5 µg/mL) showed a synergy effect with acyclovir (1 µM), leading to significant inhibition of HSV-1 infection via blocking ICP27, VP16, and gD expressions.

In a combined phytochemical profiling and antiviral study, Dias et al. [63] tested the anti-HSV-1 properties of *Arctium lappa* L. crude extract, which is rich in dibenzylbutyrolactone-type lignans arctiin and arctigenin. The crude extract at a concentration of 400 µg/mL exhibited a remarkable reduction in viral load and hence viral DNA replication. The authors suggest that the anti-HSV-1 action of the crude extract is related to the presence of arctiin and arctigenin.

Deightonin, a neolignan, has recently been obtained from the aerial parts of *Euphorbia deightonii* Croizat. with anti-herpesvirus activity. This compound has successfully inactivated the replication of HSV-2 in infected Vero cells with an IC_50_ value of 11.73 µM and SI = 3.39 [64]. 

### 3.6. Anthraquinones and Their Derivatives 

Emodin is an anthraquinone derivative distributed in various plant species, including *Aloe vera*, *Rheum palmatum*, *Polygonum multiflorum*, and *Cassia occidentalis* with proven in vitro and in vivo efficacy against HSV-1 and HSV-2 at an effective concentration of 50 µg/mL (in vitro) and doses of 3.3, 6.7, and 11.3 g/kg/day, respectively (in vivo; 7 days), according to study published in 2011 [65]. Later, in an animal experiment published in 2021 [66], several mechanisms were disclosed in which emodin was found to reduce the TLR3 pathway and its downstream molecules, TRIF, TRADD, TRAF6, traf3, Nemo, IRF3, and p38, by 20–60% as well as the expressions of IL-6 (interleukin-6), TNF-α (tumor necrosis factor-α), and IFN-β (tumor necrosis factor-β) by 30-50% in HSV-1-infected brain tissues of mice.

Mugas and colleagues [67] isolated from the aerial parts of *Heterophyllaea pustulata* seven anthraquinone-type compounds with ani-HSV-1 properties. The investigated compounds 5,5′-Bisoranjidiol, rubiadin 1-methyl ether, soranjidiol 1-methyl ether, damnacanthol, soranjidiol, rubiadin, and heterophylline suppressed the replication of HSV-1 with IC_50_ values of 15.6, 69.4, 57.1, 61.6, 27.2, 20.5, and 72.2 µM, respectively. Moreover, the test molecules possessed notable photo-inactivation (>80%) of HSV-1 particles, suggesting their potential use in treating localized herpetic lesions. 

Treatment of HSV-1-infected Vero cells with a plant-derived 1,4-Anthraquinone (6.25 µg/mL) notably impeded HSV-1 activity with a reduced value of viral titer (*Rf*; 1 × 10^2^) [68]. 

### 3.7. Curcuminoids

Curcumin is a bioactive molecule that belongs to the class of curcuminoids and has broadly been detected in diverse *Curcuma* spp. with various health benefits [69,70]. This compound at a concentration of 30 µM hindered the replication of HSV-1 and HSV-2 by blocking the adsorption of both viruses [71]. Previous work has also uncovered additional mechanisms against HSV-1 via inhibiting IE gene expression by repressing histone acetyltransferase activity of the transcriptional coactivator proteins p300 and CBP [72]. In another experiment, nanoparticle-containing curcumin (0.5 mg) decreased tissue inflammation and the severity of HSV-2 infection in animal models (female C57BL/6 mice). The mechanism of action was claimed to be linked with the anti-inflammatory properties of curcumin [73].

HSV encodes several enzymes that are necessary for viral replication, representing valuable drug targets useful for the therapy of HSV infections [29,74]. Accordingly, in an in silico assay, curcumin was studied for its capability to bind to the active site of HSV-1 TK. The results depicted that curcumin has successfully bound to HSV-1 TK active site by establishing critical molecular interactions (hydrogen bonding and hydrophobic interactions) with the amino acid residues of the enzyme active site. Hydroxyl and carbonyl groups and phenyl rings of curcumin were proposed as functional groups responsible for the anti-HSV-1 TK action [75].

Badria et al. [76], in their remarkable work, enhanced the anti-HSV-1 properties of curcumin by fabricating and optimizing a formulation of curcumin-loaded proniosomes delivery system (F5). The optimized F5 (30 µM) has notably reduced the viral plaques by 90% and hence HSV-1 replication with an acceptable level of cytotoxicity (CC_50_ = 200 µM). Also, the research team has positively determined the binding mode and molecular interaction of curcumin with HSV-1 DNA polymerase, a critical enzyme required for HSV-1 replication, by molecular docking analysis. 

### 3.8. Coumarins

Saidu and colleagues [64] have recently isolated scoparon, a coumarin type molecule, from the aerial parts of *Euphorbia deightonii* Croizat. with promising antiviral properties. Scoparon showed strong inhibitory action against HSV-2 DNA replication with an IC_50_ value of 0.032 µM and SI = 10.93.

Coumarin-type molecules such as imperatorin and phellopterin, acquired from the fruits of *Angelica archangelica* L., were noticed to be active against HSV-1 replication. Imperatorin at concentrations of 15.62 and 31.25 µg/mL reduced the titer of HSV-1 by 3.48 log and 4.7 log, respectively. Phellopterin at the concentration of 7.81 µg/mL decreased the virus titer by 3.01 log, while the mixture of imperatorin and phellopterin diminished the virus titer by 3.73 log at a concentration of 31.25 µg/mL. Direct inactivation of HSV-1 DNA replication was recorded as a mechanism of action [77].

### 3.9. Other Polyphenols: Phloroglucinol 

Okba and her research team [78] evaluated the efficacy of phloroglucinol-rich extract (PGRE) from the plant *Eucalyptus sideroxylon* A.Cunn. ex Woolls against HSV-2 infection. PGRE suppressed HSV-2 replication and attachment to Vero cells with an IC_50_ value of 189.36 µg/mL and an inhibition percentage of 87.65%. The mechanism was claimed to be attributed to impeding viral protein synthesis. 

## 4. Antiviral Properties of Non-Flavonoid Polyphenols against Beta-Herpesviruses (Human Cytomegalovirus)

HCMV is the most studied herpesvirus of the beta-herpesvirus subfamily. It causes lifelong infection in humans with the ability to replicate in leukocytes and vascular endothelial cells and further endures latently in bone marrow progenitor cells and myeloid cells [79,80]. Primary HCMV infection can be transmitted via infected saliva, blood transfusions, sex, and breastfeeding [81]. Various clinical and experimental discoveries showed that HCMV could be partially involved in the development of certain types of cancers, including nasopharyngeal carcinoma, squamous cell carcinoma, gastric cancer, glioblastoma, colorectal cancer, and breast cancer [82,83]. HCMV has also been reported to infect the nervous system and induce numerous neurological complications [84,85,86].

This section records all findings concerning non-flavonoid polyphenols and their protective effects against HCMV, with an emphasis on the mechanisms of action and pathways along with efficient concentrations or doses. However, it is important to point out that the last five years have perceived slow progress in involving non-flavonoid polyphenols in HCMV research.

### 4.1. Phenolic Acids

In a combined antiviral and multiple-biochemical study, ginkgolic acid was detected to suppress the reactivation of two HCMV clinical strains (CH19 and BI-6) in human foreskin fibroblasts cells and prevent plaque formation with IC_50_ values of 6.38 and 7.26 µM, respectively. The underlying mechanisms were revealed via inhibiting the viral entry and DNA replication [35].

### 4.2. Stilbenes 

The in vitro anti-herpesvirus effect of piceatannol on HCMV-positive human diploid fibroblast (WI-38) cells was displayed at 20 µM with diverse mechanisms of action. The mechanisms were related to blocking the expression of IE and E genes as well as the replication of HCMV DNA. Additionally, piceatannol has efficiently repressed the activation of p16^INK4a^, a key senescence-associated molecule, and cellular senescence generated by HCMV [87].

### 4.3. Anthraquinones 

During the past decade, very limited experiments have been conducted on the antiviral effect of anthraquinones against HCMV infection. Only one significant study found out that emodin could repress HCMV infection with an EC_50_ of 4.9 µM through a suggested mechanism that disturbs viral DNA replication and synthesis [88]. 

### 4.4. Curcuminoids 

The anti-HCMV properties of curcumin have previously been unveiled with diverse mechanisms, including inhibition of IEA, UL83A, IL-6, and TNF-α expressions and downregulation of heat shock protein 90 (Hsp90) [89,90]. In 2020, new mechanisms were revealed. For instance, the findings of a preclinical study verified that curcumin at concentrations of 0.5, 1, and 2 µM displayed in vitro antiviral effect on HCMV-infected endothelial cells by interfering with replication and proliferation processes. Curcumin was also observed to decrease the intracellular reactive oxygen species (ROS) overproduction, reduce the production of inflammatory cytokines, and downregulate the level of HMGB1-TLRS-NF-κB signaling pathway-related proteins. In addition to anti-HCMV activity, curcumin (at doses of 15 and 25 mg/kg/day for 10 weeks) in vivo was identified to slow down atherosclerosis progression by blocking the virus activity and HCMV-induced HMGB1-TLRS-NF-κB signaling pathway [91]. Similarly, curcumin was earlier proven to protect against HCMV infection in Balb/c mice by decreasing HCMV DNA load with a proposed mechanism that links with its anti-inflammatory and antioxidant effects [92]. 

## 5. Antiviral Properties of Non-Flavonoid Polyphenols against Gamma-Herpesviruses

Human Gamma-herpesviruses include EBV and KSHV, which are known to induce various types of cancer in humans. EBV is mainly associated with lymphomas and epithelial cell cancers and causes infectious mononucleosis (kissing disease), while KSHV generates Kaposi’s sarcoma (KS) and several B-cell cancers [93,94,95,96,97]. Both viruses can form primary, lytic, and latent infections [98]. They were also determined to be involved in infecting the nervous system, producing various neurological diseases [99,100]. The main transmission way for EBV is through infected saliva, while KSHV could be spread through several means, including saliva, blood, and sex. The primary infection of both viruses is usually asymptomatic [101,102,103].

Over the past five years, limited investigations have been performed on non-flavonoid polyphenols against gamma-herpesvirus infections. Therefore, in this section, we emphasize all available data that signify the potential role of non-flavonoid polyphenols in fighting against EBV and KSHV infections using diverse mechanisms of action at molecular levels with a focus on presenting the effective concentrations or doses. 

### 5.1. Phenolic Acids

An interesting finding was reported at the beginning of the 21st century by Nomura et al. [104] who synthetically prepared polyphenol esters consisting of two naturally occurring substances (gallic acid and ferulic acid) with anti-EBV properties. The prepared compounds exerted potent 12-*O*-tetradecanoylphorbol-13-acetate (TPA)-induced EBV activation suppression at a concentration of 20 µM in vitro.

By using the cell-cell fusion technique, ginkgolic acid (10 µM) has efficiently blocked EBV membrane fusion by hindering viral fusion glycoprotein gB, leading to blocking EBV reactivation [35]. 

EBV early antigen (EBV-EA) appears in the acute phase of EBV disease, indicating an active infection. Therefore, inhibiting EBV-EA expression represents a promising approach to preventing EBV recurrent infection and EBV-related diseases [105,106]. Consequently, Zhang et al. [107] assessed the inhibitory effect of chlorogenic acid, protocatechuic acid, and gallic acid isolated from the fruits of *Ficus hispida* L.f. against the EBV early antigen (EBV-EA) activation generated by TPA in Raji cells. The test molecules exhibited inhibitory action against EBV-EA activation with IC_50_ values of 340, 481, and 473 mol ratio/32 pmol TPA, respectively. The IC_50_ value characterizes the mol ratio of test compounds, relative to TPA, necessary to reduce 50% of the positive control stimulated with 32 pmol TPA. 

### 5.2. Stilbenes 

In KSHV-positive primary effusion lymphoma cells, resveratrol (at concentrations of 1, 5, and 20 µM) showed anti-KSHV activities through various mechanisms, including disruption of the viral latent infection, inhibition of virus lytic gene expression Rta (replication and transcription activator), and suppression of virus progeny production [108]. 

### 5.3. Lignans

The impact of manassantin B, isolated from the roots of *Saururus chinensis*, on EBV lytic replication was analyzed using P3HR-1 cells, an EBV-latently-infected Burkitt lymphoma cell line. The virus reactivation was stimulated with TPA and sodium butyrate (NaB). Manassantin B blocked the EBV lytic DNA replication and virion production with an IC_50_ value of 1.72 µM and an EC_50_ value of 0.5 µM, respectively. The mechanisms underlying the anti-EBV action were defined via inhibiting the EBV immediate-early gene BZLF1 expression by interfering with activator protein-1 (AP-1) signal transduction as well as repressing the mammalian target of rapamycin complex 2 (mTORC2)-mediated phosphorylation of AKT Ser/Thr (serine/threonine) protein kinase at Ser-473 and protein kinase Cα (PKCα) at Ser-657. The results also concluded that mTORC2 signaling plays a vital role in EBV reactivation [109].

### 5.4. Anthraquinones 

Emodin at concentrations ranging from 1 to 50 µM, inhibited the expression of EBV lytic proteins and suppressed virion production in EBV- positive epithelial cell lines via mechanisms that interfere with SP1 (transcription factor) Zta (an immediate-early gene), and Rta expressions. It exhibited tumorigenic properties against EBV-positive human cell lines (HONE-1, HA, TW01, and NA) with 50% cytotoxic concentration (CC_50_) values of 58, 65, 31, and 79 µM, respectively. Also, it repressed EBV-induced tumor growth in mice via impeding EBV reactivation at a dose of 40 mg/kg every 3 days for 4 weeks [110].

EBV nuclear antigen 1 (EBNA1) plays a vital role in the replication, partition, transcription, and protection of the viral genome, and inhibiting the expression of EBNA1 is considered a promising therapeutic target for the treatment of EBV infection [111]. Consequently, Jakhmola et al. [112] conducted docking and molecular dynamic simulations studies on EBNA1 targeting by emodin. Emodin was detected to bind to the EBNA1 active pocket with high affinity, indicating its potential use as an EBNA1 inhibitor.

An ethyl acetate subfraction of *Polygonum cuspidatum* root containing emodin (12.5 µg/mL) has successfully regulated EBV gene expression (BRLF1, BNLF1, and LMP1 (latent membrane protein 1)) and induced apoptosis of EBV-positive tumor cell during EBV reactivation stage [113]. 

At a concentration of 20 µg/mL, aloe-emodin (isolated from *Lindernia crustacea* (L.) F.Muell.) induced considerable inhibitory action against the EBV lytic cycle performed on EBV-positive Burkitt’s lymphoma P3HR1 cell line. The mechanism was ascertained by inhibiting Rta expression [114].

### 5.5. Curcuminoids 

EBV-positive nasopharyngeal carcinoma cells (such as HONE1 and HK1 cells) were exposed to curcumin treatment at concentrations of 5 and 10 µM. The findings reveal that curcumin could suppress EBV latent and lytic replication in both infected cells by reducing the expression levels of EBV transcriptional activator BZLF1 encoded by IE gene [115]. On the other hand, another investigation published in 2021 stated that curcumin mediates cytotoxicity against EBV-infected immortalized human lymphoblastoid cells, where treatment with curcumin (5 µM for 9 h) induced cell death in a concentration-dependent manner. This study explained the chemosensitizing effect of curcumin based on its ability to regulate the EBV life cycle by decreasing the level of viral protein EBNA1, leading to cell cycle arrest and apoptosis of EBV-positive cancerous cells [116].

Inactivation of recurrent KSHV infection in primary effusive lymphoma cells by curcumin was determined at a concentration of 30 µM. Curcumin has substantially blocked the virus reactivation by decreasing expression of the switch gene Rta and the delayed-early gene K8. Furthermore, it reduced intracellular and extracellular KSHV genomic DNA levels with an IC_50_ value of 8.76 µM and an EC_50_ value of 6.68 µM, respectively. It also diminished the replication of KSHV by inhibiting apurinic/apyrimidinic endonuclease 1 (APE1)-mediated redox function [117]. 

### 5.6. Coumarins and Furanocoumarins 

Inhibition of EBV-EA expression activated with TPA in Raji cells by 7-hydroxycoumarin, 7-hydroxy-6-[2-(*R*)-hydroxy-3-methyl-but-3-enyl]-hydroxycoumarin, and psoralen (a furanocoumarin-type compound) acquired from the fruits of *Ficus hispida* L.f. was effectively determined with IC_50_ values of 451, 467, and 497 mol ratio/32 pmol TPA, respectively. The IC_50_ value defines the mol ratio of test compounds, relative to TPA, needed to diminish 50% of the positive control stimulated with 32 pmol TPA [107]. 

(+)-Rutamarin, a furanocoumarin obtained from *Ruta graveolens*, has earlier been described to induce inhibitory effects on EBV lytic DNA replication and virion production with an IC_50_ value of 2.38 µM and an EC_50_ value 2.94 µM, respectively [118]. Later, this compound was also observed to hinder EBV lytic DNA replication with an IC_50_ value of 7.0 µM by a mechanism that targets viral protein synthesis [119]. (+)-Rutamarin inhibitory activities against KSHV lytic DNA replication and virion production have also been reported with an IC_50_ value of 1.12 µM and an EC_50_ value of 1.62 µM, respectively [120]. 

## 6. Mechanisms of Action of Non-Flavonoid Polyphenols: A Focus Insight

**Figure 2:**
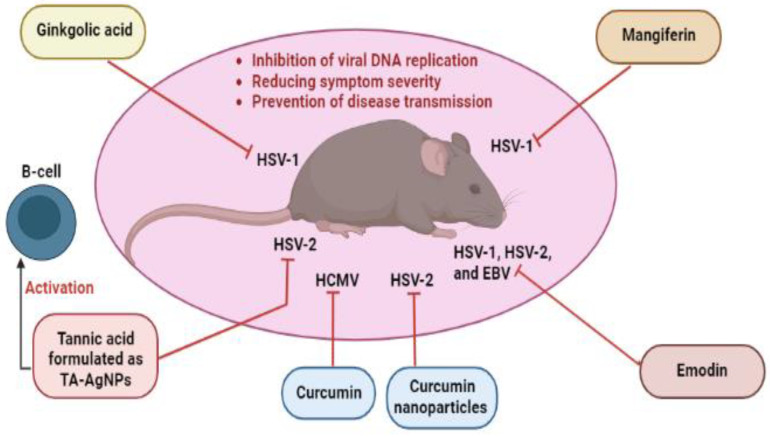
Non-flavonoid polyphenols with anti-herpesvirus properties validated by animal experiments. EBV, Epstein–Barr virus; HCMV, human cytomegalovirus; HSV-1, herpes simplex virus type 1; HSV-2; herpes simplex virus type 2; TA-AgNPs, tannic acid-modified silver nanoparticles.

**Figure 3:**
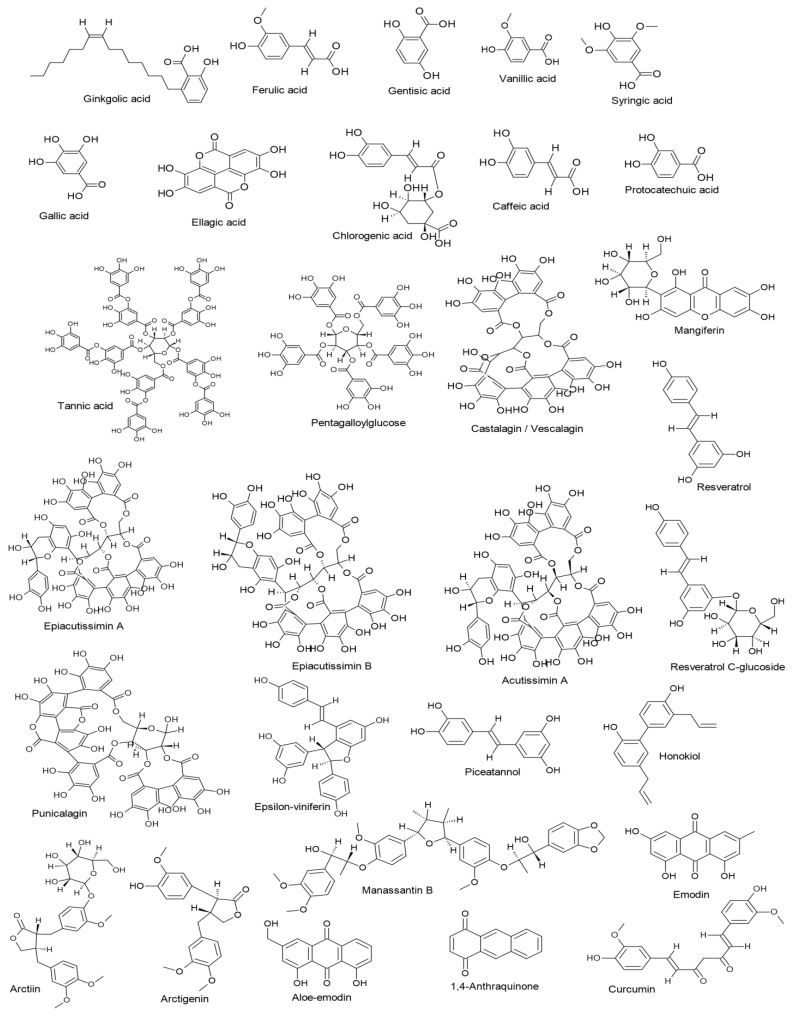

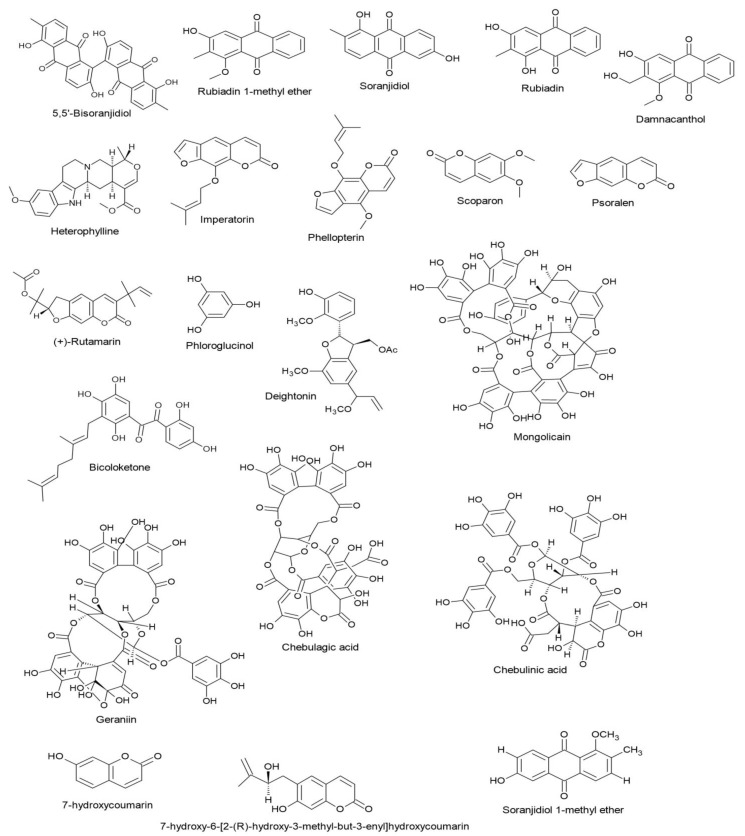
Chemical structures of the reviewed non-flavonoid polyphenols.

Targeting different stages of the herpesvirus life cycle is an effective way to eliminate the virus and its related complications. Specifically, DNA replication, a multi-step process, is an essential step of the virus life cycle and represents an appealing target for antiviral treatments [4,121,122]. Therefore, and based on the data extracted from the reviewed articles introduced in this paper, we summarize in Table 1 all mechanisms and pathways by which non-flavonoid polyphenols cause antiviral actions by interfering with multiple stages during the herpesvirus life cycle. As seen in the table, the documented compounds were detected to affect the herpesvirus DNA replication by diverse mechanisms at molecular and cellular levels via targeting viral glycoproteins, viral gene expression, viral protein synthesis, viral enzymes, NF-κB activity, and B cells activation. In addition to the impact on viral DNA replication, they were found to interfere with other steps in the viral life cycle, including attachment, entry, latency, and reactivation. The mechanisms of action of ginkgolic acid, tannic acid, mangiferin, emodin, and curcumin have been validated in animal experiments and, therefore, deserve special attention to entering clinical studies (Figure 2). The chemical structures of all reviewed compounds are shown in Figure 3 except 1,2,3,4,5-penta-*O*-digalloyl-*β*-*D*-glucopyranose and 1,2,3,4,5-penta-*O*-digalloyl-*α*-*D*-glucopyranose, where both compounds share the molecular formula C_76_H_52_O_46_. The anti-HSV-1 activity of curcumin via targeting TK was found to be linked with functional groups such as hydroxyl and carbonyl groups and phenyl rings.

## 7. Conclusions and Take-Home Message

There is no doubt that treatment of HHV infection and its complications is challenging, and new antiviral medications are urgently required. Polyphenols are one of the best naturally occurring molecules exhibiting numerous health benefits and play a crucial role as a source of natural antivirals. In this article, we showcased various chemical classes of non-flavonoid polyphenols as possible antiviral agents for treating HHV infections with diverse mechanisms at different molecular and cellular levels along with effective concentrations or doses. The reviewed compounds were evaluated by various biochemical, virological, and computational methods performed on multiple cells (animal and human cells) and animal models (mice). Compounds such as castalagin, vescalagin, and honokiol demonstrated excellent ability to interact synergistically with acyclovir, showing improved anti-HSV properties and hence enhanced treatment effectiveness. Additionally, the application of the nano-drug delivery system has shown significant efficacy in improving the anti-HSV properties of tannic acid and ellagic acid formulated in Ag-NPs and ZnO-NPs, respectively. The anti-infectivity of curcumin against HSV-1 and HSV-2 infections has also been improved by curcumin-loaded proniosomes and curcumin nanoparticles, respectively. However, more studies on nano-drug delivery combined with pharmacokinetic and pharmacodynamic assessments should be developed to enhance HHV therapy. Another therapeutic approach was achieved by employing a chelation strategy in which metal chelates of caffeic acid (addition of cations such as Fe^3+^ and anionic molecules such as molybdate and phosphate) showed significant improvement in antiviral activity against HSV infections. Besides, co-administration of zinc ions with punicalagin was observed to potentiate viricidal activity against HSV infections. The chemical synthesis of polyphenols to produce esters consisting of ferulic and gallic acids has potently increased the anti-infectivity properties against EBV. Although the safety profile of dietary polyphenols (resveratrol and curcumin) is high, their intake should be included in special diet plans, and further animal and clinical investigations should be conducted regarding their antiviral effects on HHVs. 

Finally, the presented information in this paper might provide a valuable platform for other researchers to build upon and aid them design and synthesize novel and potent compounds proper for developing anti-herpesvirus drugs that could enter clinical studies.

## Figures and Tables

**Table 1:** A focus insight into the reviewed non-flavonoid polyphenols, summarizing their mechanisms of action against human alpha-, beta-, and gamma-herpesviruses.

Compound	Chemical Class	Herpesvirus	Mechanisms of Action (Inhibition/Downregulation)
Ginkgolic acid	Phenolic acids	HSV-1, HCMV, and EBV	HSV-1 DNA replication, viral structure, ICP27, ICP8, US11, and viral progeny production. HCMV entry and its DNA replication. EBV membrane fusion and gB.
Trans-ferulic acid, gentisic acid, vanillic acid, syringic acid, and gallic acid	Phenolic acids	HSV-1 and EBV	HSV-1 DNA polymerase, HSV-1 gB (by vanillic acid), and EBV-EA (by gallic acid).
Polyphenol esters consisting of gallic acid and ferulic acid	Phenolic acids	EBV	EBV reactivation.
Ellagic acid	Phenolic acids	HSV-2	HSV-2 DNA replication.
Chlorogenic acid and caffeic acid	Phenolic acids	HSV-1 and EBV	HSV-1 gB and EBV-EA (by chlorogenic acid).
Caffeic acid chelates	Phenolic acids	HSV-1 and HSV-2	Enhancement of anti-HSV activity by inhibiting viral DNA replication and viral attachment.
Protocatechuic acid	Phenolic acids	HSV-2 and EBV	HSV-2 DNA replication and virion production. EBV-EA.
Chebulagic acid and chebulinic acid	Tannins	HSV-2	HSV-2 DNA replication.
Geraniin	Tannins	HSV-1	HSV-1 gB.
Tannic acid formulated as TA-AgNPs and purified tannic acid	Tannins (gallotannins)	HSV-1 and HSV-2	HSV-1 replication, gC, and gB (purified and formulated tannic acid), HSV-2 replication and improving the anti-HSV-2 immune response by activating B cells.
1,2,3,4,5-penta-*O*-digalloyl-*β*-*D*-glucopyranose, 1,2,3,4,5-penta-*O*-digalloyl-*α*-*D*-glucopyranose, and *α*/*β*-3-*O*-digalloyl-*D*-glucopyranose (1:1 mixture).	Tannins (gallotannins)	HSV-1	HSV-1 replication and viral glycoproteins.
Pentagalloylglucose	Tannins (gallotannins)	VZV	VZV replication, VZV-induced JNK, and VZV-IE62.
Castalagin and vescalagin	Tannins (ellagitannins)	HSV-1 and HSV-2	In combination with acyclovir, notable inhibition of HSV-1 and HSV-2 replications was observed.
Epiacutissimin B, epiacutissimin A, acutissimin A, and mongolicain	Tannins (ellagitannins)	HSV-1	HSV-1 DNA replication and viral glycoproteins.
Punicalagin	Tannins (ellagitannins)	HSV-2	HSV-2 DNA replication. HSV-2 protease.
Mangiferin	Xanthones	HSV-1	HSV-1 DNA replication and virus particles.
Resveratrol	Stilbenes	HSV-1, HSV-2, and KSHV	HSV-1 and HSV-2 replications, viral IE, and CDK9. KSHV latent infection, Rta, and formation of virus progeny.
Greco extract contains resveratrol C-glucoside, resveratrol, and epsilon-viniferin	Stilbenes	HSV-1	HSV-1 particles and viral DNA replication.
Piceatannol	Stilbenes	HSV-1, HSV-2, and HCMV	HSV-1 and HSV-2 replications and viral particles. HCMV replication, IE, E, and p16^INK4a^.
Bicoloketone	Stilbenes	HSV-1	HSV-1 DNA replication.
Honokiol	Lignans	HSV-1	HSV-1 DNA replication, ICP27, VP16, and gD.
*Arctium lappa* L. extract (rich in arctiin and arctigenin)	Lignans	HSV-1	Viral load and HSV-1 DNA replication.
Manassantin B	Lignans	EBV	EBV lytic DNA replication, virion production, BZLF1, AP-1, and mTORC2-mediated phosphorylation of AKT Ser/Thr at Ser-473 and PKCα at Ser-657.
Deightonin	Neolignans	HSV-2	HSV-2 DNA replication.
Emodin	Anthraquinones	HSV-1, HSV-2, HCMV, and EBV	HSV-1 and HSV-2 replications, TLR3 pathway and its downstream molecules (TRIF, TRADD, TRAF6, traf3, Nemo, IRF3, and p38), IL-6, TNF-α, and IFN-β. HCMV DNA replication and synthesis. EBV lytic proteins, virion production, SP1, Zta, Rta, EBNA1, BRLF1, BNLF1, and LMP1.
Aloe-emodin	Anthraquinones	EBV	EBV lytic cycle and Rta.
5,5′-Bisoranjidiol, rubiadin 1-methyl ether, soranjidiol 1-methyl ether, damnacanthol, soranjidiol, rubiadin, and heterophylline	Anthraquinones	HSV-1	HSV-1 DNA replication and HSV-1 particles (photo-inactivation).
1,4-Anthraquinone	Anthraquinones	HSV-1	HSV-1 DNA replication.
Curcumin	Curcuminoids	HSV-1, HSV-2, HCMV, EBV, and KSHV	HSV-1 and HSV-2 replications and their adsorption, HSV-1 TK, HSV-1 IE, p300, CBP and HSV-1 DNA polymerase. HCMV (IEA, UL83A, IL-6, TNF-α, Hsp90, ROS, inflammatory cytokines, HMGB1-TLRS-NF-κB). Protection against HCMV by inducing anti-inflammatory and antioxidant activities. EBV (latent and lytic replication, BZLF1, and EBNA1. KSHV (Rta, K8, and APE1-mediated redox function).
Imperatorin and phellopterin	Coumarins	HSV-1	HSV-1 DNA replication.
Scoparon	Coumarins	HSV-2	HSV-2 DNA replication.
7-hydroxycoumarin and 7-hydroxy-6-[2-(R)-hydroxy-3-methyl-but-3-enyl]hydroxycoumarin	Coumarins	EBV	EBV-EA.
Psoralen	Furanocoumarins	EBV	EBV-EA.
(+)-Rutamarin	Furanocoumarins	EBV and KSHV	EBV (lytic DNA and viral protein synthesis). KSHV (lytic DNA replication and virion production).
Phloroglucinol-rich extract (PGRE)	Other polyphenols (phloroglucinol)	HSV-2	HSV-2 DNA replication and viral protein synthesis.

Abbreviations: AKT Ser/Thr, serine/threonine protein kinase; AP-1, activator protein-1; APE1, apurinic/apyrimidinic endonuclease 1; BNLF1, viral gene; BRLF1, a transcriptional activator; BZLF1, an immediate-early gene (also known as Zta); CBP, transcriptional coactivator protein; CDK9, cyclin-dependent kinase 9; DNA, deoxyribonucleic acid; EBNA1, EBV nuclear antigen 1; EBV, Epstein–Barr virus; EBV-EA, EBV early antigen; gB, viral glycoprotein; gC, viral glycoprotein; gD, viral glycoprotein; Greco, grape cane extract from *Vitis vinifera* L.; HCMV, human cytomegalovirus; HSV-1, herpes simplex virus type 1; HSV-2, herpes simplex virus type 2; Hsp90, heat shock protein 90; ICP27, immediate early; ICP8, early protein; IE, an immediate-early; IFN-β, tumor necrosis factor-β; IL-6, interleukin-6; JNK, c-Jun N-terminal kinase; K8, delayed-early gene; KSHV, Kaposi sarcoma-associated herpesvirus; LMP1, latent membrane protein 1; mTORC2, mammalian target of rapamycin complex 2; NF-κB, nuclear factor-κB; p300, transcriptional coactivator protein; p16INK4a, a key senescence-associated molecule; PGRE, phloroglucinol-rich extract from the *Eucalyptus sideroxylon* A.Cunn. ex Woolls; PKCα, protein kinase Cα; ROS, reactive oxygen species; Rta, replication and transcription activator; SP1, transcription factor; TA-AgNPs, tannic acid combined with silver nanoparticles; TK, thymidine kinase; TNF-α, tumor necrosis factor-α; US11, late protein; VP16, a transcriptional activator; VZV, varicella-zoster virus; Zta, an immediate-early gene. An important note: This table highlights the reviewed compounds, their chemical classes, and the mechanisms of action against human herpesviruses, and the corresponding references are shown in the textual descriptions throughout the article.

## Data Availability

Not applicable.

## References

[1] Šudomová M., Berchová-Bímová K., Mazurakova A., Šamec D., Kubatka P., Hassan S.T.S. (2022). Flavonoids Target Human Herpesviruses That Infect the Nervous System: Mechanisms of Action and Therapeutic Insights. Viruses.

[2] Šudomová M., Berchová-Bímová K., Marzocco S., Liskova A., Kubatka P., Hassan S.T.S. (2021). Berberine in Human Oncogenic Herpesvirus Infections and Their Linked Cancers. Viruses.

[3] Treml J., Gazdová M., Šmejkal K., Šudomová M., Kubatka P., Hassan S.T.S. (2020). Natural Products-Derived Chemicals: Breaking Barriers to Novel Anti-HSV Drug Development. Viruses.

[4] Adler B., Sattler C., Adler H. (2017). Herpesviruses and Their Host Cells: A Successful Liaison. Trends Microbiol..

[5] Šudomová M., Hassan S.T.S. (2021). Nutraceutical Curcumin with Promising Protection against Herpesvirus Infections and Their Associated Inflammation: Mechanisms and Pathways. Microorganisms.

[6] Wołącewicz M., Becht R., Grywalska E., Niedźwiedzka-Rystwej P. (2020). Herpesviruses in Head and Neck Cancers. Viruses.

[7] Azab W., Osterrieder K. (2017). Initial Contact: The First Steps in Herpesvirus Entry. Adv. Anat. Embryol. Cell Biol..

[8] Connolly S.A., Jardetzky T.S., Longnecker R. (2021). The Structural Basis of Herpesvirus Entry. Nat. Rev. Microbiol..

[9] Cohen J.I. (2020). Herpesvirus Latency. J. Clin. Investig..

[10] Wu Y., Yang Q., Wang M., Chen S., Jia R., Yang Q., Zhu D., Liu M., Zhao X., Zhang S. (2021). Multifaceted Roles of ICP22/ORF63 Proteins in the Life Cycle of Human Herpesviruses. Front. Microbiol..

[11] Frappier L. (2015). Regulation of Herpesvirus Reactivation by Host MicroRNAs. J. Virol..

[12] Dochnal S.A., Francois A.K., Cliffe A.R. (2021). De Novo Polycomb Recruitment: Lessons from Latent Herpesviruses. Viruses.

[13] Jarosinski K.W. (2017). Interindividual Spread of Herpesviruses. Adv. Anat. Embryol. Cell Biol..

[14] Poole C.L., James S.H. (2018). Antiviral Therapies for Herpesviruses: Current Agents and New Directions. Clin. Ther..

[15] Majewska A., Mlynarczyk-Bonikowska B. (2022). 40 Years after the Registration of Acyclovir: Do We Need New Anti-Herpetic Drugs?. Int. J. Mol. Sci..

[16] Kłysik K., Pietraszek A., Karewicz A., Nowakowska M. (2020). Acyclovir in the Treatment of Herpes Viruses—A Review. Curr. Med. Chem..

[17] Hassan S.T.S., Masarčíková R., Berchová K. (2015). Bioactive Natural Products with Anti-Herpes Simplex Virus Properties. J. Pharm. Pharmacol..

[18] Hassan S.T.S., Švajdlenka E., Berchová-Bímová K. (2017). *Hibiscus sabdariffa* L. and Its Bioactive Constituents Exhibit Antiviral Activity against HSV-2 and Anti-Enzymatic Properties against Urease by an ESI-MS Based Assay. Molecules.

[19] Lattanzio V., Ramawat K.G., Mérillon J.-M. (2013). Phenolic Compounds: Introduction. Natural Products.

[20] Wang X., Qi Y., Zheng H. (2022). Dietary Polyphenol, Gut Microbiota, and Health Benefits. Antioxidants.

[21] Durazzo A., Lucarini M., Souto E.B., Cicala C., Caiazzo E., Izzo A.A., Novellino E., Santini A. (2019). Polyphenols: A Concise Overview on the Chemistry, Occurrence, and Human Health. Phytother. Res..

[22] Tuladhar P., Sasidharan S., Saudagar P. (2021). Role of Phenols and Polyphenols in Plant Defense Response to Biotic and Abiotic Stresses. Biocontrol Agents and Secondary Metabolites.

[23] Cory H., Passarelli S., Szeto J., Tamez M., Mattei J. (2018). The Role of Polyphenols in Human Health and Food Systems: A Mini-Review. Front. Nutr..

[24] Di Lorenzo C., Colombo F., Biella S., Stockley C., Restani P. (2021). Polyphenols and Human Health: The Role of Bioavailability. Nutrients.

[25] Luca S.V., Macovei I., Bujor A., Miron A., Skalicka-Woźniak K., Aprotosoaie A.C., Trifan A. (2020). Bioactivity of Dietary Polyphenols: The Role of Metabolites. Crit. Rev. Food Sci. Nutr..

[26] Zhang L., Han Z., Granato D. (2021). Polyphenols in Foods: Classification, Methods of Identification, and Nutritional Aspects in Human Health. Adv. Food Nutr. Res..

[27] Chojnacka K., Skrzypczak D., Izydorczyk G., Mikula K., Szopa D., Witek-Krowiak A. (2021). Antiviral Properties of Polyphenols from Plants. Foods.

[28] Montenegro-Landívar M.F., Tapia-Quirós P., Vecino X., Reig M., Valderrama C., Granados M., Cortina J.L., Saurina J. (2021). Polyphenols and Their Potential Role to Fight Viral Diseases: An Overview. Sci. Total Environ..

[29] Hassan S.T.S., Šudomová M., Berchová-Bímová K., Šmejkal K., Echeverría J. (2019). Psoromic Acid, a Lichen-Derived Molecule, Inhibits the Replication of HSV-1 and HSV-2, and Inactivates HSV-1 DNA Polymerase: Shedding Light on Antiherpetic Properties. Molecules.

[30] Zhu S., Viejo-Borbolla A. (2021). Pathogenesis and Virulence of Herpes Simplex Virus. Virulence.

[31] Gershon A.A., Breuer J., Cohen J.I., Cohrs R.J., Gershon M.D., Gilden D., Grose C., Hambleton S., Kennedy P.G.E., Oxman M.N. (2015). Varicella Zoster Virus Infection. Nat. Rev. Dis. Prim..

[32] Kennedy P.G.E., Gershon A.A. (2018). Clinical Features of Varicella-Zoster Virus Infection. Viruses.

[33] Azab W., Dayaram A., Greenwood A.D., Osterrieder N. (2018). How Host Specific Are Herpesviruses? Lessons from Herpesviruses Infecting Wild and Endangered Mammals. Annu. Rev. Virol.

[34] Lum K.K., Cristea I.M. (2021). Host Innate Immune Response and Viral Immune Evasion During Alphaherpesvirus Infection. Curr. Issues Mol. Biol..

[35] Borenstein R., Hanson B.A., Markosyan R.M., Gallo E.S., Narasipura S.D., Bhutta M., Shechter O., Lurain N.S., Cohen F.S., Al-Harthi L. (2020). Ginkgolic Acid Inhibits Fusion of Enveloped Viruses. Sci. Rep..

[36] Sochocka M., Sobczyński M., Ochnik M., Zwolińska K., Leszek J. (2019). Hampering Herpesviruses HHV-1 and HHV-2 Infection by Extract of Ginkgo Biloba (EGb) and Its Phytochemical Constituents. Front. Microbiol..

[37] Bhutta M.S., Shechter O., Gallo E.S., Martin S.D., Jones E., Doncel G.F., Borenstein R. (2021). Ginkgolic Acid Inhibits Herpes Simplex Virus Type 1 Skin Infection and Prevents Zosteriform Spread in Mice. Viruses.

[38] Todorova N., Rangelov M., Dincheva I., Badjakov I., Enchev V., Markova N. (2022). Potential of Hydroxybenzoic Acids from Graptopetalum Paraguayense for Inhibiting of Herpes Simplex Virus DNA Polymerase–Metabolome Profiling, Molecular Docking and Quantum-Chemical Analysis. Pharmacia.

[39] AbouAitah K., Allayh A.K., Wojnarowicz J., Shaker Y.M., Swiderska-Sroda A., Lojkowski W. (2021). Nanoformulation Composed of Ellagic Acid and Functionalized Zinc Oxide Nanoparticles Inactivates DNA and RNA Viruses. Pharmaceutics.

[40] Di Sotto A., Di Giacomo S., Amatore D., Locatelli M., Vitalone A., Toniolo C., Rotino G.L., Lo Scalzo R., Palamara A.T., Marcocci M.E. (2018). A Polyphenol Rich Extract from Solanum Melongena L. DR2 Peel Exhibits Antioxidant Properties and Anti-Herpes Simplex Virus Type 1 Activity In Vitro. Molecules.

[41] Langland J., Jacobs B., Wagner C.E., Ruiz G., Cahill T.M. (2018). Antiviral Activity of Metal Chelates of Caffeic Acid and Similar Compounds towards Herpes Simplex, VSV-Ebola Pseudotyped and Vaccinia Viruses. Antivir. Res..

[42] Kesharwani A., Polachira S.K., Nair R., Agarwal A., Mishra N.N., Gupta S.K. (2017). Anti-HSV-2 Activity of Terminalia Chebula Retz Extract and Its Constituents, Chebulagic and Chebulinic Acids. BMC Complement. Altern. Med..

[43] Siqueira E.M.D.S., Lima T.L., Boff L., Lima S.G., Lourenço E.M., Ferreira É.G., Barbosa E.G., Machado P.R., Farias K.J., Ferreira L.D.S. (2020). Antiviral Potential of Spondias Mombin L. Leaves Extract Against Herpes Simplex Virus Type-1 Replication Using In Vitro and In Silico Approaches. Planta Med..

[44] Szymańska E., Orłowski P., Winnicka K., Tomaszewska E., Bąska P., Celichowski G., Grobelny J., Basa A., Krzyżowska M. (2018). Multifunctional Tannic Acid/Silver Nanoparticle-Based Mucoadhesive Hydrogel for Improved Local Treatment of HSV Infection: In Vitro and In Vivo Studies. IJMS.

[45] Orłowski P., Kowalczyk A., Tomaszewska E., Ranoszek-Soliwoda K., Węgrzyn A., Grzesiak J., Celichowski G., Grobelny J., Eriksson K., Krzyzowska M. (2018). Antiviral Activity of Tannic Acid Modified Silver Nanoparticles: Potential to Activate Immune Response in Herpes Genitalis. Viruses.

[46] Vilhelmova-Ilieva N., Jacquet R., Deffieux D., Pouységu L., Sylla T., Chassaing S., Nikolova I., Quideau S., Galabov A.S. (2019). Anti-Herpes Simplex Virus Type 1 Activity of Specially Selected Groups of Tannins. Drug Res..

[47] Vilhelmova-Ilieva N., Jacquet R., Quideau S., Galabov A.S. (2014). Ellagitannins as Synergists of ACV on the Replication of ACV-Resistant Strains of HSV 1 and 2. Antivir. Res..

[48] Arunkumar J., Rajarajan S. (2018). Study on Antiviral Activities, Drug-Likeness and Molecular Docking of Bioactive Compounds of Punica Granatum L. to Herpes Simplex Virus-2 (HSV-2). Microb. Pathog..

[49] Houston D.M.J., Bugert J.J., Denyer S.P., Heard C.M. (2017). Potentiated Virucidal Activity of Pomegranate Rind Extract (PRE) and Punicalagin against Herpes Simplex Virus (HSV) When Co-Administered with Zinc (II) Ions, and Antiviral Activity of PRE against HSV and Aciclovir-Resistant HSV. PLoS ONE.

[50] Bae S., Kim S.Y., Do M.H., Lee C.H., Song Y.-J. (2017). 1,2,3,4,6-Penta-O-Galloyl-ß-D-Glucose, a Bioactive Compound in Elaeocarpus Sylvestris Extract, Inhibits Varicella-Zoster Virus Replication. Antivir. Res..

[51] Rechenchoski D.Z., Agostinho K.F., Faccin-Galhardi L.C., Lonni A.A.S.G., da Silva J.V.H., de Andrade F.G., Cunha A.P., Ricardo N.M.P.S., Nozawa C., Linhares R.E.C. (2020). Mangiferin: A Promising Natural Xanthone from Mangifera Indica for the Control of Acyclovir - Resistant Herpes Simplex Virus 1 Infection. Bioorg. Med. Chem..

[52] Rechenchoski D.Z., Samensari N.L., Faccin-Galhardi L.C., de Almeida R.R., Cunha A.P., Ricardo N.M.P.S., Nozawa C., Linhares R.E.C. (2019). The Combination of Dimorphandra Gardneriana Galactomannan and Mangiferin Inhibits Herpes Simplex and Poliovirus. Curr. Pharm. Biotechnol..

[53] Abba Y., Hassim H., Hamzah H., Noordin M.M. (2015). Antiviral Activity of Resveratrol against Human and Animal Viruses. Adv. Virol..

[54] Chen X., Song X., Zhao X., Zhang Y., Wang Y., Jia R., Zou Y., Li L., Yin Z. (2022). Insights into the Anti-Inflammatory and Antiviral Mechanisms of Resveratrol. Mediat. Inflamm..

[55] Docherty J.J., Fu M.M., Stiffler B.S., Limperos R.J., Pokabla C.M., DeLucia A.L. (1999). Resveratrol Inhibition of Herpes Simplex Virus Replication. Antivir. Res..

[56] Annunziata G., Maisto M., Schisano C., Ciampaglia R., Narciso V., Tenore G.C., Novellino E. (2018). Resveratrol as a Novel Anti-Herpes Simplex Virus Nutraceutical Agent: An Overview. Viruses.

[57] Ding L., Jiang P., Xu X., Lu W., Yang C., Zhou P., Liu S. (2020). Resveratrol Promotes HSV-2 Replication by Increasing Histone Acetylation and Activating NF-ΚB. Biochem. Pharmacol..

[58] Xiao J., Wang X., Wu Y., Zhao Q., Liu X., Zhang G., Zhao Z., Ning Y., Wang K., Tan Y. (2019). Synergistic Effect of Resveratrol and HSV-TK/GCV Therapy on Murine Hepatoma Cells. Cancer Biol. Ther..

[59] Zheng Y., Yang X.-W., Schols D., Mori M., Botta B., Chevigné A., Mulinge M., Steinmetz A., Schmit J.-C., Seguin-Devaux C. (2021). Active Components from Cassia Abbreviata Prevent HIV-1 Entry by Distinct Mechanisms of Action. Int. J. Mol. Sci..

[60] Tarbeeva D.V., Krylova N.V., Iunikhina O.V., Likhatskaya G.N., Kalinovskiy A.I., Grigorchuk V.P., Shchelkanov M.Y., Fedoreyev S.A. (2022). Biologically Active Polyphenolic Compounds from Lespedeza Bicolor. Fitoterapia.

[61] Squillaci G., Zannella C., Carbone V., Minasi P., Folliero V., Stelitano D., Cara F.L., Galdiero M., Franci G., Morana A. (2021). Grape Canes from Typical Cultivars of Campania (Southern Italy) as a Source of High-Value Bioactive Compounds: Phenolic Profile, Antioxidant and Antimicrobial Activities. Molecules.

[62] Liu S., Li L., Tan L., Liang X. (2019). Inhibition of Herpes Simplex Virus-1 Replication by Natural Compound Honokiol. Virol. Sin..

[63] Dias M.M., Zuza O., Riani L.R., de Faria Pinto P., Pinto P.L.S., Silva M.P., de Moraes J., Ataíde A.C.Z., de Oliveira Silva F., Cecílio A.B. (2017). In Vitro Schistosomicidal and Antiviral Activities of Arctium Lappa L. (Asteraceae) against Schistosoma Mansoni and Herpes Simplex Virus-1. Biomed. Pharmacother..

[64] Saidu M.B., Kúsz N., Tsai Y.-C., Vágvölgyi M., Berkecz R., Kókai D., Burián K., Hohmann J., Rédei D. (2022). Triterpenes and Phenolic Compounds from Euphorbia Deightonii with Antiviral Activity against Herpes Simplex Virus Type-2. Plants.

[65] Xiong H.-R., Luo J., Hou W., Xiao H., Yang Z.-Q. (2011). The Effect of Emodin, an Anthraquinone Derivative Extracted from the Roots of Rheum Tanguticum, against Herpes Simplex Virus in Vitro and in Vivo. J. Ethnopharmacol..

[66] Huang Y., Li X., Pan C., Cheng W., Wang X., Yang Z., Zheng L. (2021). The Intervention Mechanism of Emodin on TLR3 Pathway in the Process of Central Nervous System Injury Caused by Herpes Virus Infection. Neurol. Res..

[67] Mugas M.L., Marioni J., Martinez F., Aguilar J.J., Cabrera J.L., Contigiani M.S., Konigheim B.S., Núñez-Montoya S.C. (2021). Inactivation of Herpes Simplex Virus by Photosensitizing Anthraquinones Isolated from Heterophyllaea Pustulata. Planta Med..

[68] Roa-Linares V.C., Miranda-Brand Y., Tangarife-Castaño V., Ochoa R., García P.A., Castro M.Á., Betancur-Galvis L., San Feliciano A. (2019). Anti-Herpetic, Anti-Dengue and Antineoplastic Activities of Simple and Heterocycle-Fused Derivatives of Terpenyl-1,4-Naphthoquinone and 1,4-Anthraquinone. Molecules.

[69] Soleimani V., Sahebkar A., Hosseinzadeh H. (2018). Turmeric (Curcuma Longa) and Its Major Constituent (Curcumin) as Nontoxic and Safe Substances: Review. Phytother. Res..

[70] Kotha R.R., Luthria D.L. (2019). Curcumin: Biological, Pharmaceutical, Nutraceutical, and Analytical Aspects. Molecules.

[71] Flores D.J., Lee L.H., Adams S.D. (2016). Inhibition of Curcumin-Treated Herpes Simplex Virus 1 and 2 in Vero Cells. AiM.

[72] Kutluay S.B., Doroghazi J., Roemer M.E., Triezenberg S.J. (2008). Curcumin Inhibits Herpes Simplex Virus Immediate-Early Gene Expression by a Mechanism Independent of P300/CBP Histone Acetyltransferase Activity. Virology.

[73] Vitali D., Bagri P., Wessels J.M., Arora M., Ganugula R., Parikh A., Mandur T., Felker A., Garg S., Kumar M.N.V.R. (2020). Curcumin Can Decrease Tissue Inflammation and the Severity of HSV-2 Infection in the Female Reproductive Mucosa. IJMS.

[74] Xie Y., Wu L., Wang M., Cheng A., Yang Q., Wu Y., Jia R., Zhu D., Zhao X., Chen S. (2019). Alpha-Herpesvirus Thymidine Kinase Genes Mediate Viral Virulence and Are Potential Therapeutic Targets. Front. Microbiol..

[75] El-Halim S.M.A., Mamdouh M.A., El-Haddad A.E., Soliman S.M. (2020). Fabrication of Anti-HSV-1 Curcumin Stabilized Nanostructured Proniosomal Gel: Molecular Docking Studies on Thymidine Kinase Proteins. Sci. Pharm..

[76] Badria F.A., Abdelaziz A.E., Hassan A.H., Elgazar A.A., Mazyed E.A. (2020). Development of Provesicular Nanodelivery System of Curcumin as a Safe and Effective Antiviral Agent: Statistical Optimization, In Vitro Characterization, and Antiviral Effectiveness. Molecules.

[77] Rajtar B., Skalicka-Woźniak K., Świątek Ł., Stec A., Boguszewska A., Polz-Dacewicz M. (2017). Antiviral Effect of Compounds Derived from Angelica Archangelica L. on Herpes Simplex Virus-1 and Coxsackievirus B3 Infections. Food Chem. Toxicol..

[78] Okba M.M., El Gedaily R.A., Ashour R.M. (2017). UPLC-PDA-ESI-QTOF-MS Profiling and Potent Anti-HSV-II Activity of Eucalyptus Sideroxylon Leaves. J. Chromatogr. B Analyt. Technol. Biomed. Life Sci..

[79] Fulkerson H.L., Nogalski M.T., Collins-McMillen D., Yurochko A.D. (2021). Overview of Human Cytomegalovirus Pathogenesis. Methods Mol. Biol..

[80] O’Connor C.M. (2021). Cytomegalovirus (CMV) Infection and Latency. Pathogens.

[81] Griffiths P., Baraniak I., Reeves M. (2015). The Pathogenesis of Human Cytomegalovirus. J. Pathol..

[82] Michaelis M., Doerr H.W., Cinatl J. (2009). The Story of Human Cytomegalovirus and Cancer: Increasing Evidence and Open Questions. Neoplasia.

[83] Golais F., Mrázová V. (2020). Human Alpha and Beta Herpesviruses and Cancer: Passengers or Foes?. Folia Microbiol..

[84] Griffiths P. (2004). Cytomegalovirus Infection of the Central Nervous System. Herpes.

[85] Tselis A.C. (2014). Cytomegalovirus Infections of the Adult Human Nervous System. Handb. Clin. Neurol..

[86] Zhang X.-Y., Fang F. (2019). Congenital Human Cytomegalovirus Infection and Neurologic Diseases in Newborns. Chin. Med. J. (Engl).

[87] Wang S.-Y., Zhang J., Xu X.-G., Su H.-L., Xing W.-M., Zhang Z.-S., Jin W.-H., Dai J.-H., Wang Y.-Z., He X.-Y. (2020). Inhibitory Effects of Piceatannol on Human Cytomegalovirus (HCMV) in Vitro. J. Microbiol..

[88] Alam Z., Al-Mahdi Z., Zhu Y., McKee Z., Parris D.S., Parikh H.I., Kellogg G.E., Kuchta A., McVoy M.A. (2015). Anti-Cytomegalovirus Activity of the Anthraquinone Atanyl Blue PRL. Antivir. Res..

[89] Lv Y., An Z., Chen H., Wang Z., Liu L. (2014). Mechanism of Curcumin Resistance to Human Cytomegalovirus in HELF Cells. BMC Complement. Altern. Med..

[90] Lv Y., Gong L., Wang Z., Han F., Liu H., Lu X., Liu L. (2015). Curcumin Inhibits Human Cytomegalovirus by Downregulating Heat Shock Protein 90. Mol. Med. Rep..

[91] Lv Y.-L., Jia Y., Wan Z., An Z.-L., Yang S., Han F.-F., Gong L.-L., Xuan L.-L., Ren L.-L., Zhang W. (2020). Curcumin Inhibits the Formation of Atherosclerosis in ApoE-/- Mice by Suppressing Cytomegalovirus Activity in Endothelial Cells. Life Sci..

[92] Lv Y., Lei N., Wang D., An Z., Li G., Han F., Liu H., Liu L. (2014). Protective Effect of Curcumin against Cytomegalovirus Infection in Balb/c Mice. Environ. Toxicol. Pharmacol..

[93] Möhl B.S., Chen J., Longnecker R. (2019). Gammaherpesvirus Entry and Fusion: A Tale How Two Human Pathogenic Viruses Enter Their Host Cells. Adv. Virus Res..

[94] Farrell P.J. (2019). Epstein-Barr Virus and Cancer. Annu. Rev. Pathol..

[95] Yiu S.P.T., Dorothea M., Hui K.F., Chiang A.K.S. (2020). Lytic Induction Therapy against Epstein-Barr Virus-Associated Malignancies: Past, Present, and Future. Cancers.

[96] Wen K.W., Wang L., Menke J.R., Damania B. (2021). Cancers Associated with Human Gammaherpesviruses. FEBS J..

[97] Goncalves P.H., Ziegelbauer J., Uldrick T.S., Yarchoan R. (2017). Kaposi Sarcoma Herpesvirus-Associated Cancers and Related Diseases. Curr. Opin. HIV AIDS.

[98] Ackermann M. (2006). Pathogenesis of Gammaherpesvirus Infections. Vet. Microbiol..

[99] Soldan S.S., Lieberman P.M. (2020). Epstein-Barr Virus Infection in the Development of Neurological Disorders. Drug Discov. Today Dis. Models.

[100] Jha H.C., Mehta D., Lu J., El-Naccache D., Shukla S.K., Kovacsics C., Kolson D., Robertson E.S. (2015). Gammaherpesvirus Infection of Human Neuronal Cells. mBio.

[101] Nowalk A., Green M. (2016). Epstein-Barr Virus. Microbiol. Spectr..

[102] Ciccarese G., Trave I., Herzum A., Parodi A., Drago F. (2020). Dermatological Manifestations of Epstein-Barr Virus Systemic Infection: A Case Report and Literature Review. Int. J. Dermatol..

[103] Li S., Bai L., Dong J., Sun R., Lan K., Cai Q., Yuan Z., Lan K. (2017). Kaposi’s Sarcoma-Associated Herpesvirus: Epidemiology and Molecular Biology. Infectious Agents Associated Cancers: Epidemiology and Molecular Biology.

[104] Nomura E., Hosoda A., Morishita H., Murakami A., Koshimizu K., Ohigashi H., Taniguchi H. (2002). Synthesis of Novel Polyphenols Consisted of Ferulic and Gallic Acids, and Their Inhibitory Effects on Phorbol Ester-Induced Epstein-Barr Virus Activation and Superoxide Generation. Bioorg. Med. Chem..

[105] Crowley A., Connell J., Schaffer K., Hall W., Hassan J. (2012). Is There Diagnostic Value in Detection of Immunoglobulin g Antibodies to the Epstein-Barr Virus Early Antigen?. Biores. Open Access.

[106] Boonsopon S., Maghsoudlou A., Kombo N.E., Foster C.S. (2016). A Therapeutic Trial of Valganciclovir in Patients with Uveitis and Positive Epstein-Barr Virus Early Antigen D IgG Titers. Eur. J. Ophthalmol..

[107] Zhang J., Zhu W.-F., Xu J., Kitdamrongtham W., Manosroi A., Manosroi J., Tokuda H., Abe M., Akihisa T., Feng F. (2018). Potential Cancer Chemopreventive and Anticancer Constituents from the Fruits of Ficus Hispida L.f. (Moraceae). J. Ethnopharmacol..

[108] Tang F.-Y., Chen C.-Y., Shyu H.-W., Hong S., Chen H.-M., Chiou Y.-H., Lin K.-H., Chou M.-C., Wang L.-Y., Wang Y.-F. (2015). Resveratrol Induces Cell Death and Inhibits Human Herpesvirus 8 Replication in Primary Effusion Lymphoma Cells. Chem. Biol. Interact..

[109] Wang Q., Zhu N., Hu J., Wang Y., Xu J., Gu Q., Lieberman P.M., Yuan Y. (2020). The MTOR Inhibitor Manassantin B Reveals a Crucial Role of MTORC2 Signaling in Epstein-Barr Virus Reactivation. J. Biol. Chem..

[110] Wu C.-C., Chen M.-S., Cheng Y.-J., Ko Y.-C., Lin S.-F., Chiu I.-M., Chen J.-Y. (2019). Emodin Inhibits EBV Reactivation and Represses NPC Tumorigenesis. Cancers.

[111] Dheekollu J., Wiedmer A., Ayyanathan K., Deakyne J.S., Messick T.E., Lieberman P.M. (2021). Cell-Cycle-Dependent EBNA1-DNA Crosslinking Promotes Replication Termination at OriP and Viral Episome Maintenance. Cell.

[112] Jakhmola S., Jonniya N.A., Sk M.F., Rani A., Kar P., Jha H.C. (2021). Identification of Potential Inhibitors against Epstein–Barr Virus Nuclear Antigen 1 (EBNA1): An Insight from Docking and Molecular Dynamic Simulations. ACS Chem. Neurosci..

[113] Yiu C.-Y., Chiu Y.-J., Lin T.-P. (2021). The Ethyl Acetate Subfraction of Polygonum Cuspidatum Root Containing Emodin Affect EBV Gene Expression and Induce EBV-Positive Cells Apoptosis. Biol. Pharm. Bull..

[114] Tsai Y.-C., Hohmann J., El-Shazly M., Chang L.-K., Dankó B., Kúsz N., Hsieh C.-T., Hunyadi A., Chang F.-R. (2020). Bioactive Constituents of Lindernia Crustacea and Its Anti-EBV Effect via Rta Expression Inhibition in the Viral Lytic Cycle. J. Ethnopharmacol..

[115] Liu L., Yang J., Ji W., Wang C. (2019). Curcumin Inhibits Proliferation of Epstein-Barr Virus-Associated Human Nasopharyngeal Carcinoma Cells by Inhibiting EBV Nuclear Antigen 1 Expression. Biomed. Res. Int..

[116] Martínez-Castillo M., Cruz-Robledo G., Hernández-Zavala A., Córdova E.J. (2021). Curcumin Sensitizes Epstein-Barr-Immortalized Lymphoblastoid Cell Lines to Inorganic Arsenic Toxicity. Exp. Ther. Med..

[117] Li H., Zhong C., Wang Q., Chen W., Yuan Y. (2019). Curcumin Is an APE1 Redox Inhibitor and Exhibits an Antiviral Activity against KSHV Replication and Pathogenesis. Antivir. Res..

[118] Wu T., Wang Y., Yuan Y. (2014). Antiviral Activity of Topoisomerase II Catalytic Inhibitors against Epstein-Barr Virus. Antivir. Res..

[119] Lin Y., Wang Q., Gu Q., Zhang H., Jiang C., Hu J., Wang Y., Yan Y., Xu J. (2017). Semisynthesis of (-)-Rutamarin Derivatives and Their Inhibitory Activity on Epstein-Barr Virus Lytic Replication. J. Nat. Prod..

[120] Xu B., Wang L., González-Molleda L., Wang Y., Xu J., Yuan Y. (2014). Antiviral Activity of (+)-Rutamarin against Kaposi’s Sarcoma-Associated Herpesvirus by Inhibition of the Catalytic Activity of Human Topoisomerase II. Antimicrob. Agents Chemother..

[121] Coen D.M., Lawler J.L., Abraham J. (2021). Herpesvirus DNA Polymerase: Structures, Functions, and Mechanisms. Enzymes.

[122] Piret J., Boivin G. (2021). Antiviral Drugs Against Herpesviruses. Adv. Exp. Med. Biol..

